# A standardized, open-source, portable model for noninvasive joint injury in mice

**DOI:** 10.1016/j.ocarto.2025.100679

**Published:** 2025-09-17

**Authors:** Michael D. Newton, Lindsey Lammlin, Sofia Gonzalez-Nolde, Scarlet Howser, Isabelle Smith, Luke Stasikelis, Alexander J. Knights, RE-JOIN Consortium Investigators, Armen Akopian, Kyle Allen, Alejandro Almarza, Benjamin Arenkiel, Maryam Aslam, Basak Ayaz, Yangjin Bae, Bruna Balbino de Paula, Anita Bandrowski, Mario Danilo Boada, Jacqueline Boccanfuso, Jyl Boline, Dawen Cai, Dellina Lane Carpio, Robert Caudle, Racel Cela, Yong Chen, Rui Chen, Brian Constantinescu, Yenisel Cruz-Almeida, M. Franklin Dolwick, Chris Donnelly, Zelong Dou, Joshua Emrick, Malin Ernberg, Danielle Freburg-Hoffmeister, Jeremy Friedman, Spencer Fullam, Janak Gaire, Akash Gandhi, Terese Geraghty, Benjamin Goolsby, Stacey Greene, Nele Haelterman, Zhiguang Huo, Michael Iadarola, Shingo Ishihara, Sudhish Jayachandran, Zixue Jin, Alisa Johnson, Frank Ko, Zhao Lai, Brendan Lee, Yona Levites, Carolina Leynes, Jun Li, Martin Lotz, Lindsey Macpherson, Tristan Maerz, Camilla Majano, Anne-Marie Malfait, Maryann Martone, Simon Mears, Bella Mehta, Emilie Miley, Rachel Miller, Richard Miller, Michael Newton, Alia Obeidat, Soo Oh, Merissa Olmer, Dana Orange, Miguel Otero, Kevin Otto, Folly Patterson, Marlena Pela, Daniel Perez, Sienna Perry, Theodore Price, Hernan Prieto, Russell Ray, Dongjun Ren, Margarete Ribeiro Dasilva, Alexus Roberts, Elizabeth Ronan, Oscar Ruiz, Shad Smith, Mairobys Soccorro Gonzalez, Kaitlin Southern, Joshua Stover, Michael Strinden, Hannah Swahn, Evelyne Tantry, Sue Tappan, Cristal Villalba Silva, Airam Vivanco-Estella, Robin Vroman, Joost Wagenaar, Lai Wang, Kim Worley, Joshua Wythe, Jiansen Yan, Julia Younis, Tristan Maerz

**Affiliations:** a Department of Orthopaedic Surgery, University of Michigan, Ann Arbor, MI, USA; b Institute for Biomechanics, Department of Health Sciences and Technology, ETH Zürich, Zürich, Switzerland; c Department of Orthopaedic Surgery, Washington University, St. Louis, MO, USA; d Center of Regenerative Medicine, Washington University, St. Louis, MO, USA; e Department of Biomedical Engineering, University of Michigan, Ann Arbor, MI, USA; f Division of Rheumatology, Department of Internal Medicine, University of Michigan, Ann Arbor, MI, USA

## Abstract

**Objective:**

Preclinical models of osteoarthritis (OA) are crucial for the study of disease mechanisms and development of disease-modifying therapeutics. While surgical OA models, such as destabilization of the medial meniscus (DMM), have been the gold standard in the field for decades, noninvasive joint loading-based models have increased in popularity and utility. To facilitate standardization of the noninvasive anterior cruciate ligament rupture (ACLR) model in mice, we present the **Mo**bile **J**oint-Injury **O**perator (**MoJO**) - an open-source protocol with accompanying fixtures and data, designed for a low-cost, commercially-available, portable, small-footprint uniaxial testing system.

**Methods:**

We provide 3d-printable fixture designs and a comprehensive description of the loading protocol, describe the expected mechanical output, and offer various troubleshooting strategies. We validate the mechanical accuracy and inter-operator reproducibility of the procedure and characterize the resultant post-traumatic OA phenotype by knee hyperalgesia testing, flow cytometry, μCT imaging, and histological assessment.

**Results:**

Across *n* ​= ​952 procedures, the MoJO protocol was highly accurate and repeatable, with a >99 ​% rate of successful ACLR and high repeatability between operators and institutions. ACLR-mediated joint injury resulted in the expected post-traumatic OA phenotype in male and female C57Bl/6 mice, including progressive hyperalgesia, histological and μCT evidence of cartilage damage, synovitis, and osteophyte formation, and increased expansion of fibroblasts, endothelial cells, and myeloid cells by flow cytometry.

**Conclusions:**

Increased standardization of joint injury is a critical aspect of the overall refinement of animal models of OA. The MoJO represents an affordable and highly reproducible option for implementing the mouse ACLR model of OA.

## 1 Introduction

Preclinical animal models of osteoarthritis (OA) are invaluable for the study of disease mechanisms and the early development of novel disease-modifying therapeutics. While rats, guinea pigs, dogs, sheep, pigs, goats, and horses are also employed in clinical translation pipelines, mouse models remain the most widely utilized and most economical tool for preclinical investigation.

Among the strongest risk factors for the development of OA is joint injury, such as anterior cruciate ligament rupture (ACLR). Following ACLR, long-term human clinical studies demonstrate a ∼40 ​% risk of developing post-traumatic OA (PTOA) [1], with some studies indicating as high as 80 ​% [2]. Given that the majority of human OA data has been collected at timepoints of already established disease, the early injury-induced mechanisms that initiate disease progression represent a major knowledge gap. Surgical OA mouse models are limited in their ability to assess early post-injury mechanisms given the need for surgical arthrotomy in the sham control group, which exhibits synovitis and hyperalgesia for up to 2–4 weeks post-injury [3,4].

**Fig. 1:**
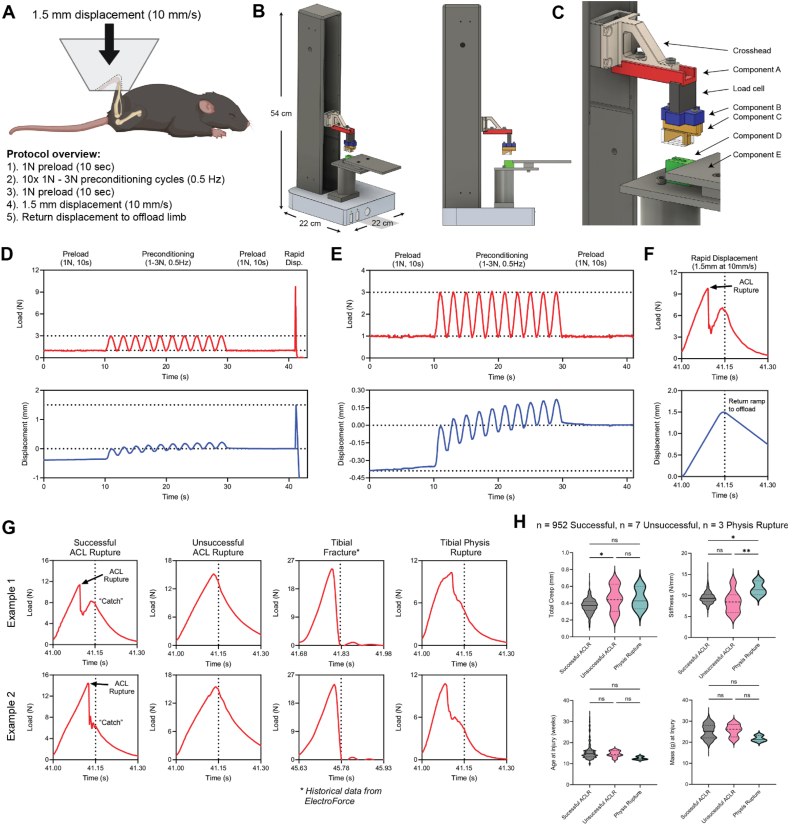
A). Schematic of mouse positioning and protocol overview. B). Isometric and side view of the Univert S2 and assembled ACLR fixtures. C). Isometric view of ACLR fixture components. D). Load vs time (top) and displacement vs time (bottom) plots of full loading protocol. E). Isolated view of load vs time (top) and displacement vs time (bottom) plots of preload and preconditioning segments to demonstrate expected creep. F). Isolated view of load vs time (top) and displacement vs time (bottom) plots of the final compressive loading segment to induce ACLR. The point of ACL failure is noted. G). Two representative examples of load vs time plots of successful and unsuccessful ACL ruptures, tibial fractures, and tibial physis ruptures. Note the characteristic secondary “catch” that should be observed following ACL rupture. H). Quantitative comparison of mechanical data, age at injury, and mass at injury between mice in successful ACLR, unsuccessful ACLR, and physis ruptures.

Noninvasive joint injury models have become increasingly popular, and they complement surgical models in their utility to assess early post-injury processes. The tibial compression overload ACLR model (Fig. 1A) is among the most widely utilized noninvasive model of PTOA in mice [[5], [6], [7], [8]] and rats [[9], [10], [11], [12]], and multiple variations have been developed, each with differences in equipment, fixtures, animal positioning, and loading protocols, resulting in a range of phenotypes. Subcritical cyclic loading has also been employed as a PTOA model with similar experimental setups [13,14]. A relatively large barrier to entry associated with noninvasive models is the need for a mechanical testing system with high displacement and load resolution. These systems are generally costly ($100,000+) and require expertise to calibrate, tune, and operate. Due to their size and/or sensitive components, they are generally immovable, greatly restricting experimental flexibility (e.g. movement in and out of vivaria), especially in multi-institutional collaborations.

To facilitate standardization and ease of access to the ACLR model in the preclinical OA community, we present an open-source protocol with accompanying fixtures and data, designed for a low-cost, commercially-available, portable uniaxial testing system. The **Mo**bile **J**oint-Injury **O**perator **(MoJO)** demonstrates a high degree of repeatability, and hyperalgesia phenotyping, cellular analyses, and histological assessments confirm the expected PTOA phenotype consistent with our prior studies employing a conventional, immobile mechanical testing system.

## 2 Methods

### 2.1 Mechanical testing system and load cell

Our protocol utilizes the Univert S2 (CellScale Inc., Waterloo, ON, Canada), a lightweight (8 ​kg), low footprint (22 ​× ​22 ​× ​54 cm), portable electromechanical testing system; however, the injury protocol and associated fixtures may be adapted to any given mechanical testing system with performance specifications similar to those of the CellScale Univert S2 (see manufacturer specifications). Given the need for high acceleration and displacement rate over a short distance, electromechanical systems are recommended, as servohydraulic systems are generally less capable of achieving target velocities over the short displacements used for mice. During the rapid compressive ramp in our protocol, the Univert S2 crosshead accelerates at a consistent 650 ​mm/s^2^, reaching the target 10 ​mm/s velocity in 14.3 ​ms (∼9.5 ​% of the total 150 ​ms displacement event) and over a 0.1 ​mm distance (∼6.7 ​% of the total distance). After reaching the target displacement, the crosshead decelerates at 604.5 ​mm/s^2^, reaching 0 ​mm/s in 18.7 ​ms (12.5 ​% of the total 150 ​ms displacement event) and over a 0.1 ​mm distance (∼6.7 ​% of the total distance).

For the utilization of this protocol in mice, a 20 ​N or 25 ​N load cell is recommended. This ensures accuracy in the preload and preconditioning segments while ensuring that the ultimate injury load, generally 9–15 ​N in C57Bl/6 mice, does not exceed the maximum load specification. The load cell utilized for our design is the Zhimin ZMSA 2 ​kg ​S beam load cell (Anhui Zhimin Electrical Technology Co., Ltd., Bengbu, China), which has a single M3 mounting screw at each end for assembly.

### 2.2 Fixtures

Our fixture design (Fig. 1B and C,Suppl.Fig. 1A-C) is comprised of a minimal list of components, all amenable to 3d printing, with standard M3 and M5 fasteners used for assembly. To minimize the chance for fixture deflection/deformation and maximize fixture lifespan, we recommend high-stiffness, high-toughness plastic for 3d printing. We employ Tough 2000 resin, printed on a Form 4B printer (Formlabs Inc., Somerville, MA, USA). The 3D designs for all components are available on the associated Github repository (https://github.com/maerz-research/mojo). Suppl.Fig.1 is a detailed component and assembly plan. In our initial implementation, the animal bed assembly (components E1-4) was machined out of aluminum stock, but our design files include models for 3D printing these parts. Additional details on fixture design and adaptation to different setups are provided in Suppl.Methods.1.

### 2.3 Mouse positioning and procedure

Following anesthetic induction, mice are placed prone on the animal bed (E1) and the knee of either hindlimb is placed over the cranial edge of the knee fixture (D) (Suppl.Fig.1D). The hindpaw is then gently manipulated into Component C, visualizing complete seating of the hindpaw into the fixture. This is most easily accomplished with two operators, one mouse handler to simultaneously guide the hindpaw and knee into their respective troughs, and a second computer operator who slowly lowers the crosshead until a low load (<0.2–0.5 ​N) is registered. Limb manipulation is easily performed with small, blunt manipulators (e.g. forceps), with one holding the knee in the trough while the other guides the hindpaw into the hindpaw fixture (C). By gently applying traction to the trunk of the mouse in the cranial direction, secure positioning of the knee in the knee trough (D) is ensured, which is confirmed via gentle medial-lateral manipulation. Appropriate mouse positioning is illustrated in Suppl.Fig.1D.

Following positioning, a downward, compressive load is applied to the joint by gradually lowering the crosshead until ∼1 ​N is reached, and the automated protocol is initiated. The injury loading protocol involves five steps (Fig. 1D):

1. 1 ​N preload for 10 ​s (load-controlled)
2. Ten sinusoidal preconditioning cycles between 1 and 3 ​N, 0.5 ​Hz (load-controlled)
3. 1 ​N preload for 10 ​s (load-controlled)
4. Injury-inducing 1.5 ​mm downward displacement, 10 ​mm/s (displacement-controlled)
5. Return displacement to offload the hindlimb, 5 ​mm upward, 5 ​mm/s (displacement-controlled)

Mice tolerate this procedure well. Healthy mice do not exhibit any post-injury weight loss or alterations to general-health indicators. Inhaled isoflurane is sufficient for anesthesia (4–5 ​% for induction, 1–2 ​% for maintenance, flow rate 0.4–0.8 ​L/min). Post-procedural analgesia may vary study-by-study. We employ carprofen (single injection, 5 ​mg/kg, subcutaneous) for unilateral ACLR, and buprenorphine XR (single injection, 3.25 ​mg/kg, subcutaneous) for bilateral ACLR. We utilize unilateral ACLR in most experiments. Bilateral ruptures produce additional pain and distress and exacerbated disease progression, and should be utilized in specific cases where (1) within-subject paired design is experimentally important, and/or (2) where their use enables substantial reduction in mice needed for a well-powered experiment, in accordance with the AAALAC 3R principles. We utilize bilateral ruptures for strictly ≤7-day post-injury endpoints.

### 2.4 Determination of successful ACL rupture

Examples of mechanical outputs from successful and unsuccessful ACL rupture procedures are shown in Fig. 1G. Successful ACL rupture can be reliably assessed via a combination of mechanical data assessment and visual/auditory cues. Successful rupture will result in an abrupt drop in load as the ACL fails and the tibia subluxes anteriorly, but *this should not result in full unloading of the joint* – a “catch” and/or secondary re-loading will generally be observed as downward displacement continues and other joint tissues (e.g. meniscus) provide resistance. For larger mice which undergo ACL rupture later in the 1.5 mm displacement stage, re-loading may not occur, but the “catch” should still be observable (Fig. 1G, Example 2). A successful ACL rupture should produce a characteristic audible “pop” that can be recognized with repetition. Anterior subluxation of the tibia, though rapid, can be visually appreciated. Successful ACL rupture can be physically assessed via the anterior drawer test [9], though in mice this procedure is challenging to interpret and should be minimized to avoid additional trauma to the joint. When first adopting the model, successful isolated ACL rupture should be confirmed via *postmortem* dissection in trial cohorts. In our hands, the success rate of this procedure is incredibly high (99.0 ​%), but unsuccessful procedures do occur. In our experience, unsuccessful ruptures produced characteristically distinct loading profiles and audiovisual feedback (Fig. 1G). Additional details for recognizing and diagnosing both successful and unsuccessful ruptures are provided in Suppl.Methods.2.

### 2.5 Mechanical tester tuning

Correct tuning is essential for load-controlled movements to execute accurately. The Univert S2 is equipped with simplified, pre-programmed velocity and acceleration tuning (scale 0–10), and we have found that values of 9 for both parameters produce excellent results. These parameters serve as simplified alternatives to full proportional–integral–derivative (PID) tuning, though advanced options do allow for further fine-tuning of PID values if desired. A protocol file for the Univert S2 control software is available on the Github repository. The tuning parameters we employ are embedded with this file. If adjustments to tuning are desired, we recommend using a steel compression spring – this should also be used to confirm correct performance at the start of each testing day, prior to live animal experimentation (see Suppl. Methods.3 for more information).

**Caution:** Changing PID parameters can cause any mechanical testing system to become unstable and oscillate at high frequency. To avoid injury, only experienced operators should perform PID tuning, and a safety shield and safety glasses should always be used during tuning.

### 2.6 Mechanical data analysis

We developed an annotated MATLAB script to automatically analyze mechanical data outputs from this protocol, available on the Github repository. Data outputs include rupture displacement, ultimate displacement, rupture load, ultimate load, linear elongation stiffness, and percent errors in preconditioning load amplitudes and ultimate displacement. These data, in addition to a graphical output of displacement vs time and load vs time plots, are important quality control tools and should be reviewed following each procedure to confirm successful ACLR. To permit prospective evaluation of protocol repeatability, our Github repository contains sample mechanical data outputs from our MoJO procedures. An analogous code has also been provided to analyze the results of a test protocol run on the calibration spring, which we recommend using to monitor the performance of the testing system over time.

### 2.7 Mechanical data validation

All C57Bl/6 mice used for validation throughout this study (*N* ​= ​27 total) were utilized under an institutional animal care and use committee (IACUC)-approved protocol, group-housed in a facility with a 12-h light/dark cycle and allowed *ad libitum* access to food and water before/after ACLR procedures. Sample sizes were determined based on previous experience with this model [8,15]. Experimental cohorts were randomized, analyses were conducted blinded to experimental conditions, and no animals included in this study were excluded from analysis. A detailed breakdown of experimental cohorts and downstream analyses is provided in Suppl.Table.1-2.

To validate the MoJO system, we performed bilateral ACLRs in C57Bl/6 mice (*N* ​= ​9) whereby one limb underwent ACLR using the MoJO system and the other limb underwent ACLR using our established platform on a conventional, immobile mechanical testing system (ElectroForce 3300AT, TA Instruments, New Castle DE, USA) [8,15,16]. Analysis of mechanical outputs were performed in paired fashion between the two limbs. Postmortem dissection and x-ray imaging, on *n* ​= ​6 mice, confirmed successful ACL rupture (with intact posterior cruciate and medial/lateral collateral ligaments) in all cases, with no x-ray evidence of femoral footprint avulsion or physeal displacement, and notable ligament tissue on both the femoral and tibial footprints, suggesting mid-substance ACL failure in all mice.

To assess the reproducibility and accuracy of the MoJO system over a large number of procedures, we retrospectively reviewed mechanical data for all mouse ACLR procedures performed within our lab to date (*N* ​= ​952), all utilized under IACUC-approval for various projects. Mice used for these procedures included males and females, and both C57Bl/6 mice (*n* ​= ​558) as well as several transgenic mouse lines (*n* ​= ​397) spanning >15 strains of global, inducible, and conditional knockouts, along with reporter strains and functionally wild-type controls.

### 2.8 Comparative flow cytometry of ACLR-induced synovial inflammation

To confirm that the MoJO protocol recapitulates the injury-induced synovitis we have characterized in the ACLR model using the conventional ElectroForce system, male C56Bl/6 mice were subjected to bilateral ACLRs (i.e. one limb via MoJO, the other via ElectroForce, *n* ​= ​3)^8, 14, 15^. At 7 days post-injury, whole knee synovium (inclusive of Hoffa's fat pad) was microdissected, digested enzymatically, as previously described [15,16], and flow cytometry was performed to assess major synovial cell populations (Suppl.Methods.4).

### 2.9 Comparative knee hyperalgesia

To confirm that the progressive knee sensitization associated with ACLR was comparable between the MoJO and ElectroForce systems, twelve mice were randomized to unilateral ACLR via either MoJO or ElectroForce systems (*n* ​= ​3/sex/system). Blinded pressure application-based knee hyperalgesia testing was performed at baseline and 7, 14, and 28 days post-ACLR, as previously described [1], using contralateral joints as controls. The average of triplicate measurements was calculated.

### 2.10 Confirmation of structural PTOA phenotype

To confirm that the MoJO system recapitulates the structural and histological features of PTOA, six mice were randomized to unilateral ACLR or an anesthesia/analgesia-only sham procedure on the MoJO system (*n* ​= ​3/condition), and were euthanized at 28 days post-injury, representing a timepoint of moderate to established disease severity. Hindlimbs were dissected immediately *post mortem,* fixed in 10 ​% neutral-buffered formalin for 48 ​h, and transferred to 70 ​% ethanol for storage. Hindlimbs were rehydrated in PBS overnight and imaged by μCT (8.9 ​μm voxel, 55 ​kVp, Bruker Skyscan). Limbs were paraffin processed, embedded, and sectioned in the sagittal plane (5 ​μm sections). Safranin-O/Fast-Green staining was used to demonstrate the expected phenotype in the medial and lateral compartments [15].

### 2.11 Statistical analyses

Statistical comparisons were performed using Prism (v10.3.0, GraphPad Software LLC, San Diego, CA, USA) and SPSS (v29.0, IBM Corp., Armonk, NY, USA). Comparisons between MoJO and ElectroForce systems in matched mechanical, flow cytometry, and hyperalgesia data were performed via one-way repeated-measures ANOVA with system as the between-subject effect. Normality and homogeneity of variances were confirmed via Shapiro-Wilk and Levene's test, respectively. Correlations of sex, age at injury, and mass at injury to mechanical parameters were performed via Pearson correlation analysis. Comparisons in mechanical data between operators and laboratories were assessed via ANCOVA, with operator or institution as the between-subject effect and age and sex as cofactors.

## 3 Results

### 3.1 Mechanical data

Analysis of mechanical data from *n* ​= ​952 MoJO ACLRs demonstrates a high degree of success and repeatability. The overall rate of successful ACL ruptures was 99.0 ​% (952/962). The ten unsuccessful procedures were comprised of 7 unsuccessful ACL ruptures and 3 tibial physeal displacements. Analysis of these cases shows no significant difference in age or body mass of the mice (Fig. 1H), but mechanical analysis shows that unsuccessful ACLRs had greater total creep during preloading and preconditioning, consistent with improper positioning (Fig. 1H). Physis ruptures had higher stiffness during linear displacement, suggesting poor positioning and engagement of the long bones rather than the ACL.

**Fig. 2:**
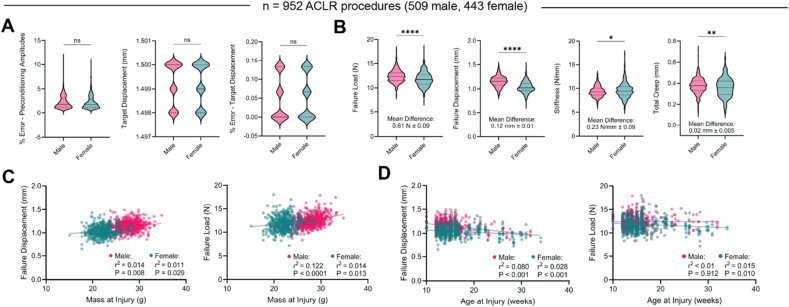
A). Accuracy and repeatability data of the ACLR protocol with the Univert S2. B). Descriptive ACLR outcomes derived from analysis of mechanical data, stratified by sex across n ​= ​509 male and n ​= ​443 female mice. C). Linear correlations between body mass at the time of injury and failure displacement (left) and failure load (right). D). Linear correlations between age at the time of injury and failure displacement (left) and failure load (right). P and r^2^ values were calculated via Pearson bivariate correlation analysis.

We observed low error in reaching target loads during preconditioning and in reaching the target displacement of the compressive rupture ramp (Fig. 2A). The total displacement during the rupture event ranged 1.498–1.500 ​mm, never exceeding the target displacement. Failure displacement, failure load, stiffness during linear elongation, and total creep also exhibit high consistency, with expected differences between male and female mice (Fig. 2B). We observed some significant but very weak linear correlations between failure load/displacement and the body mass and age of mice (Fig. 2C-D).

Comparing C57BL/6 mice (*n* ​= ​558) to transgenic strains (*n* ​= ​397) broadly, we observed no significant differences in failure load or failure displacement, with a minor but significant difference in stiffness (4.4 ​% higher in transgenic mice). Notably, transgenic mice accounted for 8/10 failed ACLRs, including all 3 physeal displacements and 5/7 unsuccessful ruptures. We attribute this finding to the varying soft tissue phenotypes in transgenic stains in our group, in addition to potential effects from tamoxifen in inducible models. Despite this, overall success rates were still high in both groups (99.6 ​% in C57BL/6, 98.0 ​% in transgenic). These data suggest the MoJO ACLR model can be successfully applied to a broad range of genotypes with musculoskeletal relevance, though genetic modifications which substantially alter the mechanical properties of bone or soft tissue might present greater challenges.

To interrogate repeatability between operators, we analyzed mechanical data between 4 experienced operators within our laboratory (*n*_*1*_ ​= ​313, *n*_2_ ​= ​289, *n*_3_ ​= ​245, *n*_4_ ​= ​108 ACLRs respectively). There were significant differences in mouse sex and age between operators, and these were statistically accounted for as cofactors (Suppl.Fig.4A). Failure load, failure displacement, and stiffness were similar between operators, with mean percent differences <3 ​% for all parameters (Suppl.Fig4B). Minor, though statistically-significant differences were detected in failure load and stiffness between operators, likely due to small differences in limb positioning coupled with high statistical power. We further compared mechanical data from our laboratory (*n* ​= ​955) to that of a collaborating laboratory (*n* ​= ​43) utilizing a separate but identical implementation of the MoJO system (Suppl.Fig.5A). Again, we found comparable mechanical outcomes between institutions (Suppl.Fig.5B), with only minor differences in failure displacement and stiffness (Suppl.Fig.5C).

These findings confirm the highly repeatable, reproducible, and accurate nature of this model. Nevertheless, each group should develop their own quality control processes with respect to acceptable mechanical outcomes, ideally corroborated by gross dissection to confirm ACL rupture and to rule out unexpected injuries in initial cohorts.

### 3.2 Comparison to ElectroForce system

**Fig. 3:**
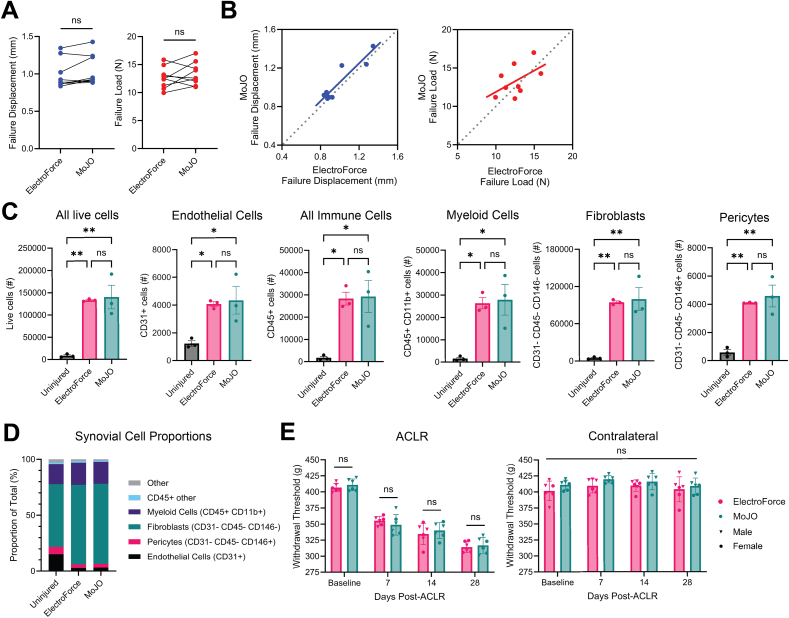
A). Failure displacement (left) and failure load (right) in a cohort of matched ACLR procedures in which the ElectroForce protocol was employed on one limb and the MoJO protocol on the other (N ​= ​9 mice). B). Linear correlations between ElectroForce- and MoJO-derived failure displacement (left) and failure load (right). C). Flow cytometric assessment of synovial cells in naïve/uninjured mice, ElectroForce-injured mice, and MoJO-injured mice. n ​= ​3 samples per condition, each made up of two pooled synovia. D). Synovial cell proportions derived from flow cytometry between uninjured, ElectroForce-injured, and MoJO-injured mice. E). Knee withdrawal thresholds from ACLR joints (left) and contralateral joints (right), derived from blinded longitudinal knee hyperalgesia testing in ElectroForce-injured and MoJO-injured mice. n ​= ​6 mice per group assessed longitudinally.

In a comparative cohort of bilateral ACLRs, in which one limb underwent ACLR with the MoJO system and the other limb underwent ACLR with our conventional ElectroForce system, we found a high degree of repeatability between the two systems and protocols (Fig. 3), confirming that the MoJO protocol recapitulated the conventional protocol. There were no differences in failure displacement or failure load at the point of ACLR between matched MoJO-injured and ElectroForce-injured joints (Fig. 3A), and these outcomes were linearly correlated between the two limbs (Fig. 3B).

As a readout of ACLR-induced synovitis, we performed flow cytometric analysis of bilateral (i.e. MoJO on one limb vs ElectroForce on the other limb) synovial tissue 7 days following ACLR, employing sham mice as uninjured controls. We observed the expected increases in synovial endothelial, immune, and stromal cells in both ACLR groups, with no significant differences in absolute cell number (per single synovium) of live cells or any subset between the two systems (Fig. 3C). The injury-induced shift in cell proportions between uninjured and ACLR synovia was highly similar between MoJO and ElectroForce (Fig. 3D), consistent with our previous characterizations of the phenotypic diversification in the immune [17] and stromal cells [16] underpinning ACLR-induced synovitis.

To confirm that the two models induce similar alterations in pain behavior, we performed blinded longitudinal knee hyperalgesia testing in separate cohorts of age- and sex-matched mice injured unilaterally with the two systems. Following injury, we observed the expected reductions in knee withdrawal thresholds in ACLR joints, with no differences in withdrawal threshold at baseline or any post-ACLR timepoint between MoJO and ElectroForce (Fig. 3E).

Taken together, these data demonstrate that the MoJO system accurately recapitulates our conventional protocol using the ElectroForce system, with equivalent mechanical induction of the injury, similar alterations in synovial cell populations, and highly comparable onset of knee hyperalgesia.

### 3.3 Structural PTOA phenotype

**Fig. 4:**
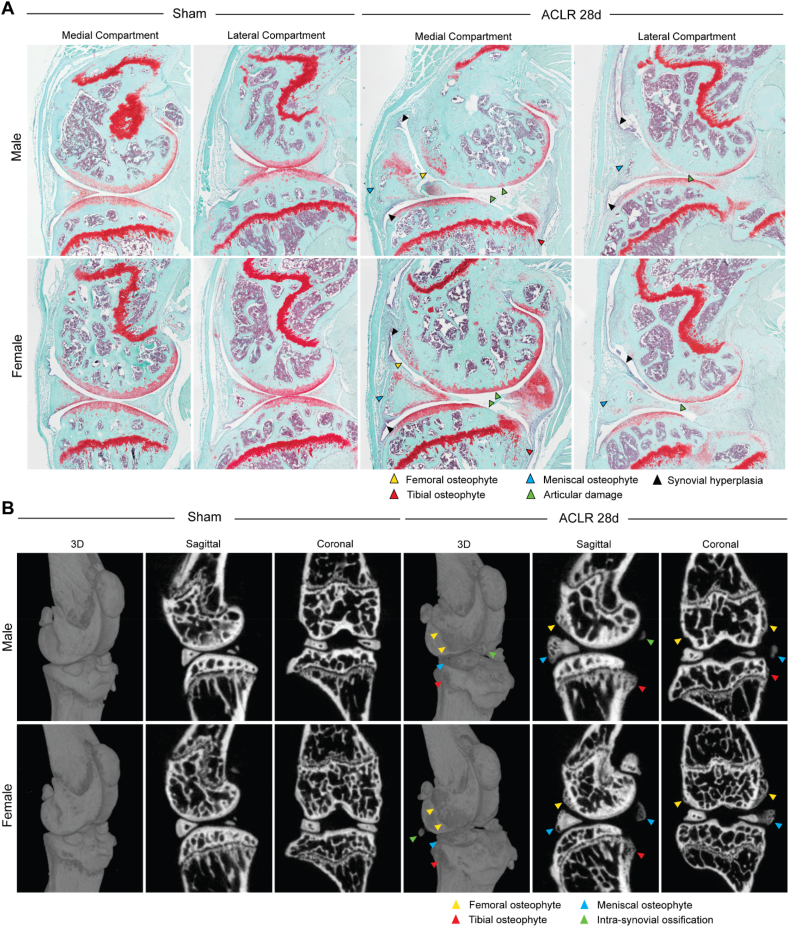
A). Safranin-O/Fast-green staining of sagittal sections from the medial and lateral compartments of Sham and ACLR 28d mice. B). 3D, sagittal, and coronal views of whole joints, derived from μCT imaging. Expected phenotypic characteristics are labeled with arrowheads.

To illustrate the expected PTOA phenotype using the MoJO system, we performed qualitative histological and μCT imaging in Sham or ACLR 28d mice. Consistent with our prior sex-dependent characterization of this model using our conventional ElectroForce protocol [15], we observed the expected onset of articular and meniscal damage, the formation of osteophytes and chondrophytes, most prominently at the anteromedial meniscus, and subchondral bone sclerosis (Fig. 4A). Articular damage was most severe at the anterior aspect of the medial femoral condyle and the posterior aspect of the medial tibial plateau, where we observed complete erosion of noncalcified cartilage down to calcified cartilage, and in severe examples, down to subchondral bone (Fig. 4A). Articular damage is milder in the lateral compartment, where we observed superficial articular damage but no massive lesions or complete erosion. Osteophyte formation is also milder in the lateral compartment.

The synovial phenotype was also consistent with our prior published work, characterized by synovial lining hyperplasia, sub-synovial fibrosis, immune cell infiltrate, volumetric loss of Hoffa's fat pad, and synovial exudate (Fig. 4A). In the posterior synovium, focal cartilage formation was observed at varying degrees (potentially also involving a remnant portion of the posterior meniscal horn), and synovial pannus was occasionally identified as invading and covering anterior tibial plateau cartilage (Fig. 4A). Male mice exhibit worse articular damage and synovitis in this model, as previously characterized [15].

μCT imaging illustrates the formation of osteophytes and chondrophytes at the anterior meniscus, the anterior femur, and the posterior tibia (Fig. 4B), highly characteristic of this model. These can be contoured in 3D to derive quantitative osteophyte formation metrics, which we've shown to vary between male and female mice [15]. In addition to osteophyte formation, intra-synovial ossification may also be observed (Fig. 4B).

## 4 Discussion

We present a comprehensive open-source protocol with associated quality control-enabling analysis tools and data to operationalize the murine ACLR model with a relatively low-cost, commercially-available, low footprint, mobile mechanical testing system. Comparative mechanical data outputs demonstrate strong parity with a conventional implementation using a high-cost ElectroForce instrument, and mechanical data in *n* ​= ​952 MoJO ACLRs demonstrates high repeatability and success rate. Male and female mice exhibited the expected induction of knee hyperalgesia and synovial cell changes (comparable with the ElectroForce implementation), and at later post-ACLR timepoints we observed phenotypic hallmarks of murine PTOA including osteophyte formation, synovial fibrosis and articular damage, demonstrating successful adaptation of the mouse ACLR model of PTOA from the ElectroForce system to the Univert S2 system.

The increasing availability of lower-cost, portable testing systems has made mechanical injury models like ACLR more accessible than ever before, particularly for laboratories without considerable mechanical testing experience. Despite the markedly lower cost, the MoJO platform executed the mouse ACLR protocol consistently and with high mechanical accuracy comparable to a considerably more expensive system. While accuracy and consistency will vary by make and model, generally the smaller footprint of these systems is an advantage, as they are inherently designed to operate at smaller length scales relevant to rodent models. In addition, the portability of the system enables easy transport between rooms, laboratories, and even institutions, greatly increasing opportunities for collaboration, cross-training, rigor and repeatability, and study design flexibility.

The MoJO platform should be considered in context with the broader benefits and drawbacks of ACLR as a preclinical PTOA model (Table .1). The protocol is rapid, repeatable, and well-tolerated by mice, and produces rapid, progressive structural and pain-relevant changes (early changes by 3-7d post-injury with established PTOA by 28d [8,15,16]). As a noninvasive model with a clinically-relevant injury mechanism, ACLR is a uniquely well-suited model for studying the acute injury responses of joint tissues; in particular, the absence of a surgical incision to the synovium makes ACLR especially advantageous for the study of synovial biology, and our comparative flow cytometry and histological data confirms this facet of the ACLR model is recapitulated accurately on the MoJO. Another major advantage is the availability of both sham and contralateral joints as controls. Sham mice receive a single anesthetic cycle and analgesia dose, exhibit no nociceptive or tissue-level changes, and are effectively naïve, healthy controls where both limbs may be utilized. Contralateral joints undergo altered gait following ipsilateral joint injury [18], but do not exhibit injury-induced joint damage, synovitis, osteophyte formation, or changes to the synovial transcriptome compared to uninjured Sham mice, based on our characterization up to 28 days post-injury [15].

**Table 1:** Advantages and Disadvantages of ACLR compared to other preclinical models of PTOA.

***Advantages***	
Entirely noninvasive	- Not categorized as surgical procedure for IACUC
	- Doesn't require sham procedures as control
Highly repeatable	
Accurate induction of clinically-relevant joint injury	- Recapitulates motion profile of non-contact, sports-related ACL injury9,14
Rapid protocol	- ∼5 ​min per mouse
Good tolerability	- No weight loss
	- No obvious signs of ill health or high pain
Straightforward, logical controls	- Contralateral and/or sham joints
Ability to assess early post-injury timepoints	- No confounding due to surgical approach
Useful for studying pain-correlated PTOA manifestations	- Synovitis, knee hyperalgesia, osteophyte formation
Useful for studying sex differences	- Observed sexual dimorphism in phenotypic presentation1
	-Female mice also exhibit PTOA development
***Disadvantages***	
Rapid onset/progression of articular damage; high endpoint severity	- Scientists interested in studying milder articular cartilage and meniscus damage should consider the DMM or PMx models
Persistent joint instability	- May make implantation of chondral treatments challenging
Overlapping phases of post-injury inflammation and established PTOA	- May make treatment effects and timing difficult to determine or interpret
Concomicant meniscal damage/tears may be unwanted in specific studies	- Scientists interested in preserving the meniscus should consider the surgical ACL transection model

Our implementation of ACLR utilizes a 1.5 ​mm displacement and 10 ​mm/s displacement rate. In our data, ACL rupture occurred at 0.9–1.3 ​mm in ∼90 ​% of mice. We target 1.5 ​mm to ensure that >99 ​% of mice exhibit complete ACL rupture, as our standard protocol does not allow re-loading of a failed rupture, with failed ruptures necessitating euthanasia; thus, the additional 0.2–0.6 ​mm of post-rupture displacement is a safeguard against incomplete/failed rupture to minimize mouse utilization, consistent with the 3R principles. Some groups may wish to employ a lower target displacement of 1.25 or 1.3 ​mm, either when working with smaller mice or to avoid over-subluxation of the joint. Histological analysis demonstrates that in this model, anterior subluxation of the posterior meniscus can occur, which may be associated with displacement beyond ACL failure and could drive greater articular damage. This subluxation is presumably progressive [8], though acute injury-induced subluxation is possible.

Despite its considerable utility in studying PTOA initiation and progression, the persistent joint instability and aggressive onset of articular destruction result in a more severe phenotype in the ACLR model compared to other commonly-utilized mouse models, such as DMM or PMx. Nonetheless, it is likely possible to modulate the severity of this model, either by reducing displacement below 1.5 ​mm in the setting of a complete rupture, or by substantially decreasing displacement to induce subcritical injuries. By reducing loading, phenotypic manifestations such as articular damage and osteophytes may be mutable. While our group has not tested this, others have employed subcritical cyclic or single-cycle loading to induce PTOA in mice [13,14,19,20]. Further, low-level cyclic tibial compression was shown to exhibit therapeutic effects in DMM-induced OA [21], while surgical restabilization of the murine joint following noninvasive ACLR was recently demonstrated, with promising evidence that restabilization can mitigate disease onset, particularly when coupled with joint unloading [22,23].

## 5 Conclusion

Mouse models will continue to be an important tool for uncovering the causal disease mechanisms of OA and PTOA while also being a part of the development and translation of novel disease-modifying treatments. To facilitate the continued standardization of models with the goal of greater reproducibility across studies and sites, this study presented an open-source protocol and validation dataset of a mobile, low-cost ACLR system.

## Author contributions

Dr. Maerz had full access to all the data included in this article and takes full responsibility for the integrity of the data and accuracy of the information. All authors have been involved in the analysis and interpretation of the data and contributed to the final manuscript.

Conception and design: Newton, Lammlin, Knights, Maerz.

Collection and assembly of data: Newton, Lammlin, Gonzalez-Nolde, Howser, Smith, Stasikelis, Knights, Maerz.

Analysis and interpretation of data: All authors.

Drafting of article: Newton, Lammlin, Stasikelis, Knights, Maerz.

Critical revision of the article for important intellectual content: All authors.

Obtaining of funding: Lammlin, Knights, Maerz.

## Disclosures

The authors have no financial conflicts related to any of the commercial entities mentioned in this study.

## Funding

This work was directly supported by the National Institute of Arthritis and Musculoskeletal and Skin Diseases (NIAMS) of the National Institutes of Health under Award Number UC2 AR082186, as part of the Restoring Joint Health and Function to Reduce Pain (RE-JOIN) consortium. TM was further supported by NIAMS (R01 AR080035, R21 AR080502, R21 AR082016, R21 AR076487), a Catalyst Award from the Dr. Ralph and Marian Falk Medical Research Trust, and the Department of Defense Congressionally Directed Medical Research Programs (CDMRP) (GRANT13696744). AJK was supported by K99 AR081894. LL was supported by a National Science Foundation Graduate Research Fellowship.

Research reported in this publication was supported by the National Institute of Arthritis and Musculoskeletal and Skin Diseases of the National Institutes of Health under Award Number P30 AR069620. The content is solely the responsibility of the authors and does not necessarily represent the official views of the National Institutes of Health.
