# Supplementary material for: A standardized, open-source, portable model for noninvasive joint injury in mice
Source: Osteoarthr Cartil Open. 2025 Sep 17;7(4):100679. doi: 10.1016/j.ocarto.2025.100679 (PMC12666826; doi:10.1016/j.ocarto.2025.100679)
Supplement: Multimedia component 1 [file mmc1.docx]

SUPPLEMENTAL INFORMATION

**A standardized, open-source, portable model for noninvasive joint injury in mice**

Michael D. Newton, Lindsey Lammlin, Sofia Gonzalez-Nolde, Scarlet Howser, Isabelle Smith, Luke Stasikelis, Alexander Knights, *Tristan Maerz

*Corresponding Author Information:

Tristan Maerz, PhD
Institute for Biomechanics, Department of Health Sciences and Technology
ETH Zürich
Gloriastrasse 39
Zürich, Switzerland
maerzt@ethz.ch

**Table of Contents:**

**Supplementary Table 1.** *Study cohorts, sample sizes, and data collected.* p. s2

**Supplementary Table 2.** *Summary of study cohorts by analysis type* p. s2

**Supplemental Figure 1.** *MoJO fixture components, assembly, and positioning* p. s3

**Supplemental Figure 2.** *Undiagnosed physeal displacement* p. s4

**Supplemental Figure 3.** *Mechanical outcomes of C57BL/6 vs. transgenic mice strains* p. s5

**Supplemental Figure 4.** *Reproducibility of ACL rupture outcomes between operators* p. s5

**Supplemental Figure 5.** *Reproducibility of ACL rupture outcomes between institutions* p. s6

**Supplemental Methods 1.** *Additional Information on Fixture Design* p. s6-s7

**Supplemental Methods 2.** *Diagnosis of Successful and Unsuccessful ACL Ruptures* p. s7-s8

**Supplemental Methods 3.** *Calibration Spring Tuning* p. s8

**Supplemental Methods 4.** *Flow Cytometry of Synovial Cell Populations* p*.* s8

**Supplementary Table 1.** *Study cohorts, sample sizes, and data collected.*


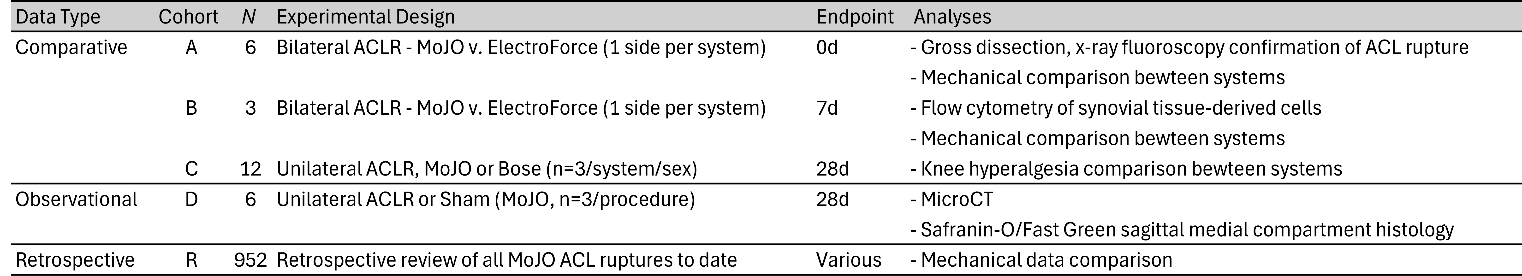


**Supplementary Table 2.** *Summary of study cohorts analysis type.*


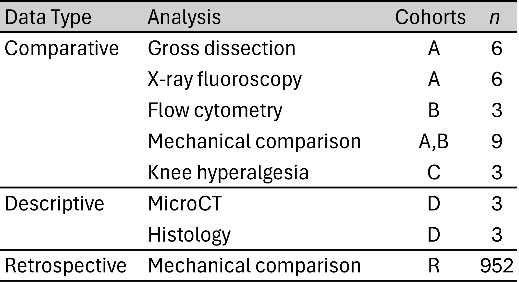


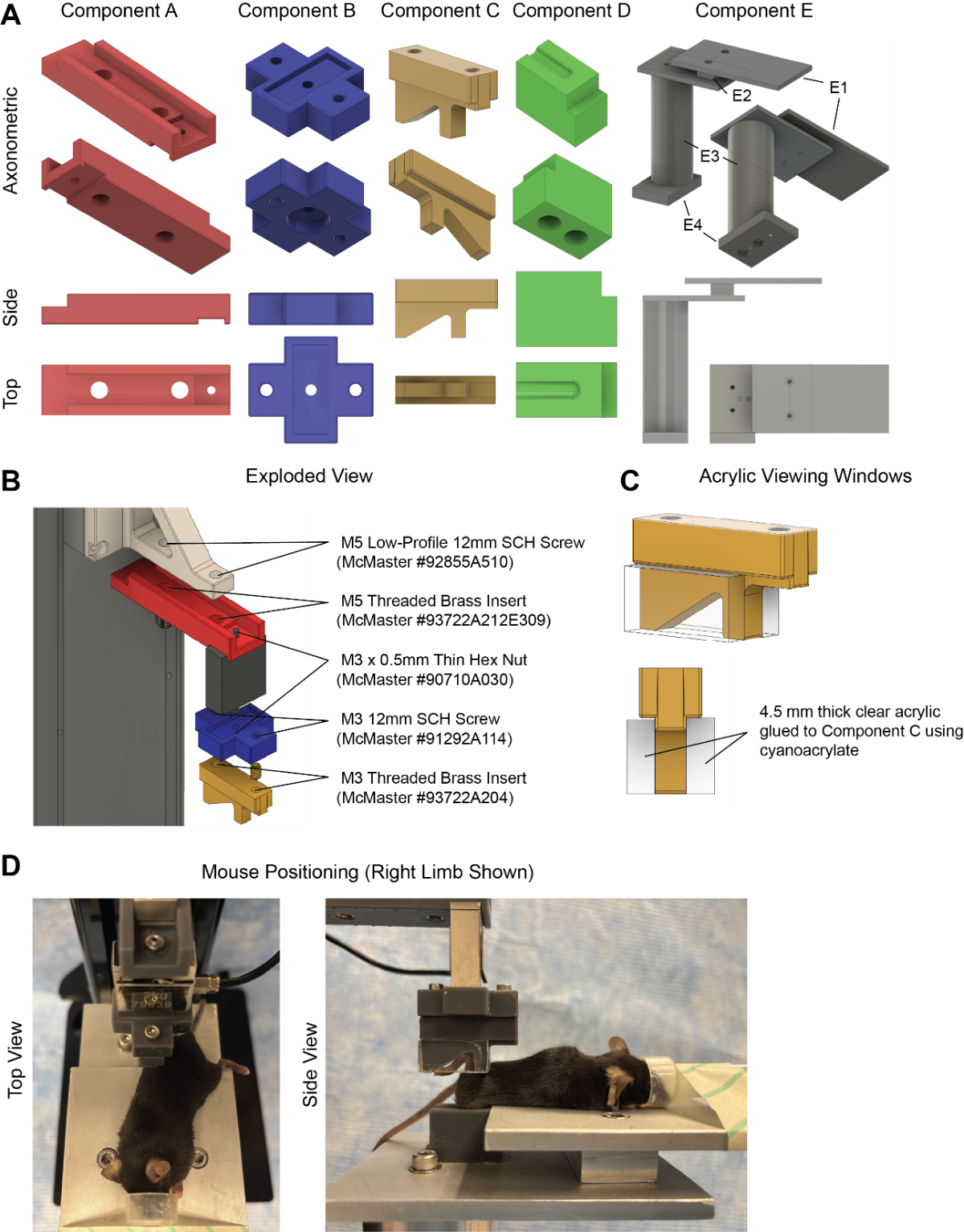


**Supplemental Figure 1 –** *MoJO fixture components, assembly, and positioning.* *(A) Isometric, side, and top views of MoJO ACL rupture fixture components A-E*. *(B) Exploded view of upper fixture assembly. Fastener locations and part numbers are denoted. (C) Installation of acrylic viewing windows on component C (hindpaw fixture). (D) Top and side views of a mouse with right limb properly positioned within the ACL rupture fixture. Note that the head is in the anesthesia nosecone, the body is fully extended, the ankle and knee are fully seated at the front of their respective holders, and that the body is positioned such that the head, hip, knee, and ankle are approximately aligned. To achieve this positioning, the contralateral hip is externally-rotated to splay the contralateral limb on the animal bed, and the body and head are slightly rotated away from true prone positioning toward the loaded limb (in this case, to the right).*


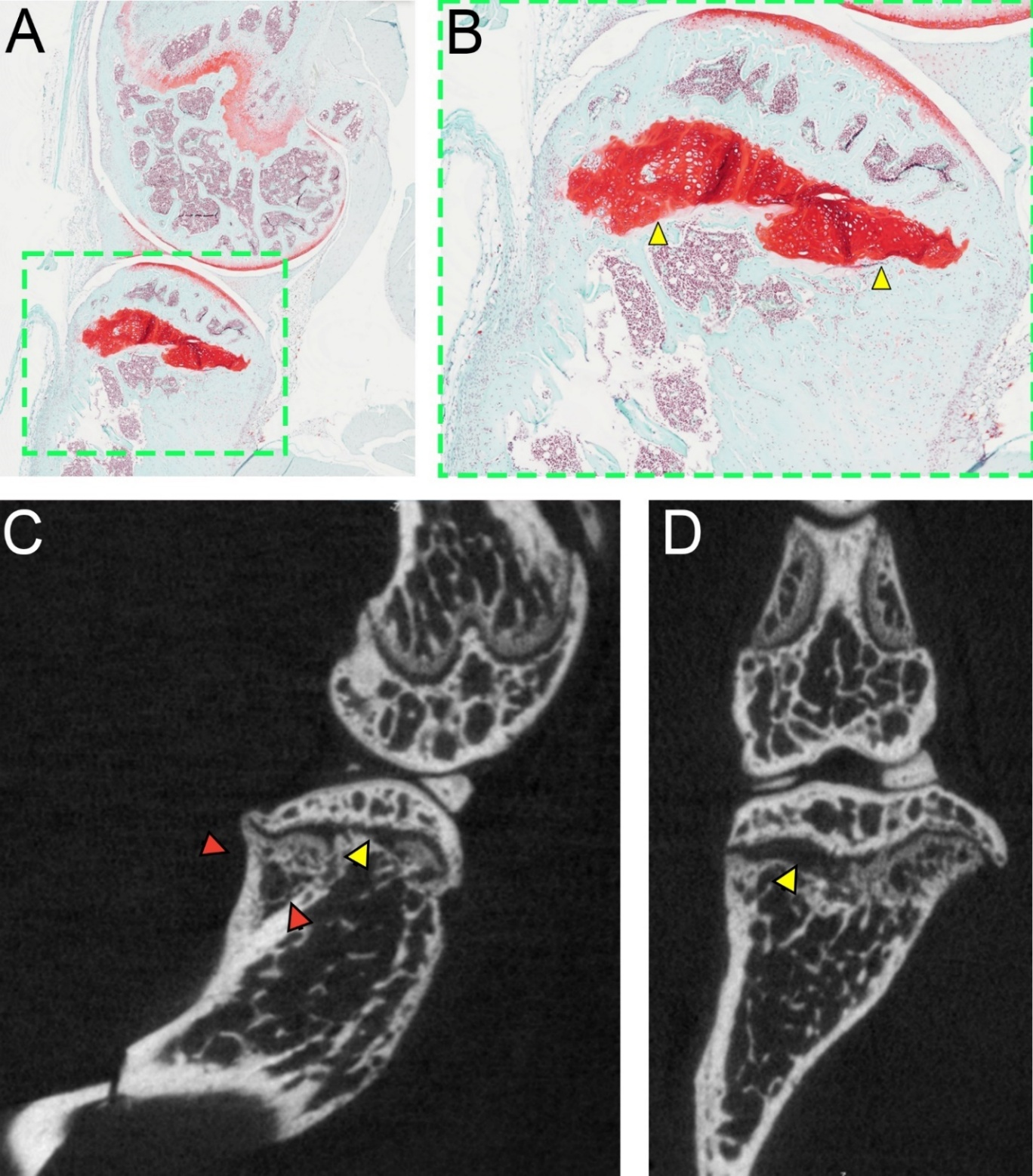


**Supplemental Figure 2 –** *Example of a healed* *physeal rupture. (A) Sagittal Safranin-O-stained histological section of the medial joint compartment, including (B) a high-magnification image of the tibial plateau, demonstrating an abnormally large physis which has been filled in by disorganized growth plate cartilage healing tissue (yellow arrows). (C) Sagittal and (D) coronal microCT sections demonstrate an enlarged tibial physis (yellow arrows), along with the presence of ectopic bone (red arrows) which appears to have formed to support a posteriorly shifted tibial epiphysis.*

*
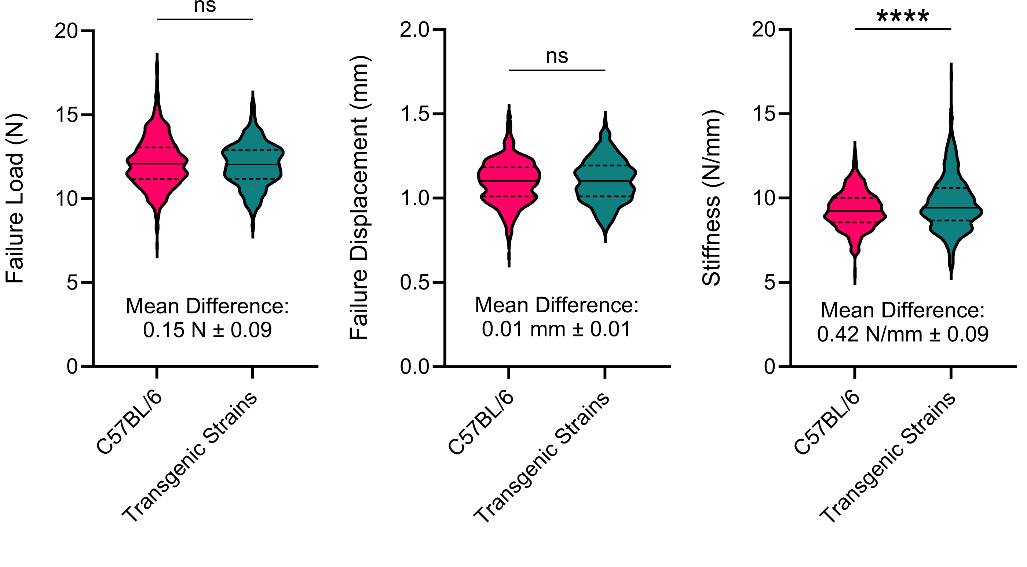
*

**Supplemental Figure 3.** *Mechanical parameters of ACL ruptures between C57BL/6 (n=558) and transgenic mouse strains (n=397), including failure load, failure displacement, and stiffness.*


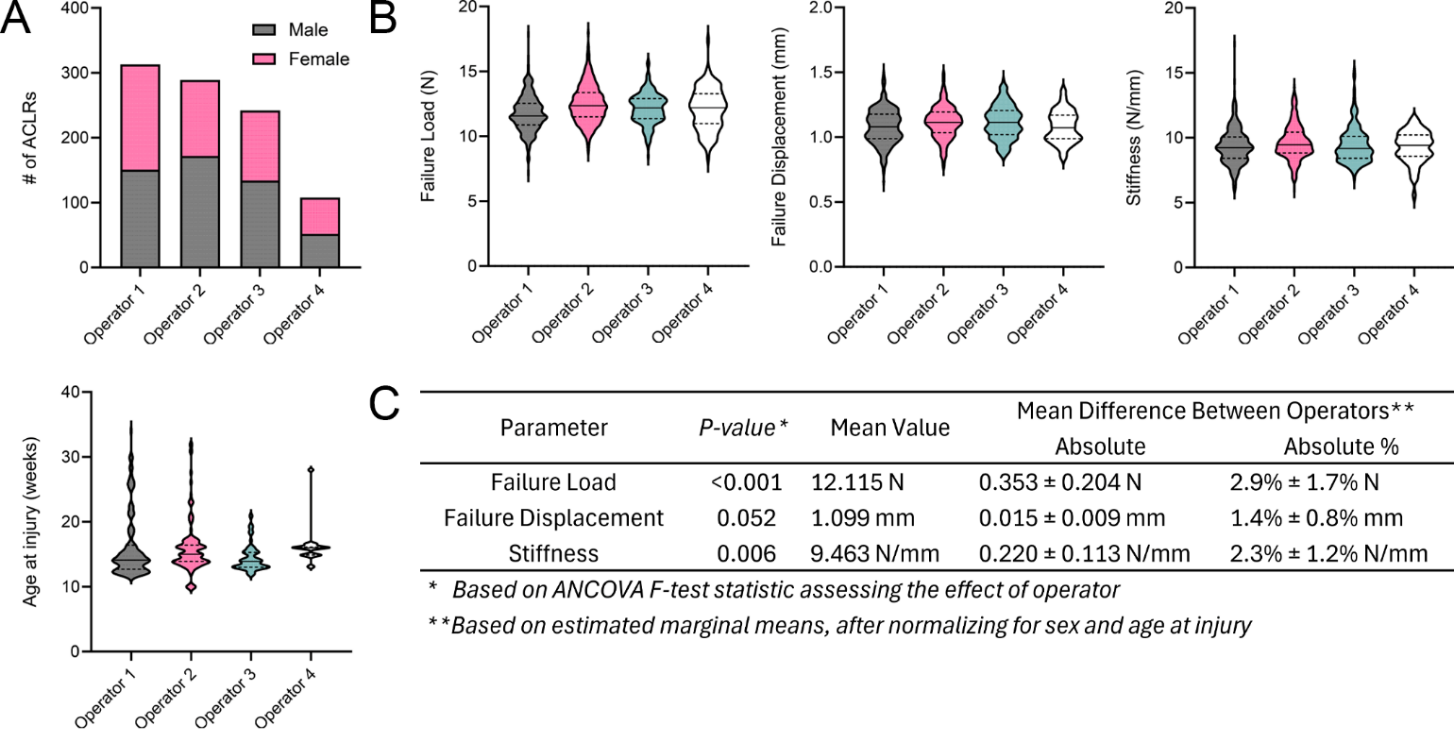


**Supplemental Figure 4.** *Reproducibility of MoJO ACL rupture outcomes between operators. (A) Distributions of sex and age at injury among 4 MoJO operators from the same laboratory. (B) Mechanical parameters of ACL ruptures by operator, including failure load, failure displacement, and stiffness. (C) Statistical analysis of differences in mechanical outcomes between operators was assessed via ANCOVA, with operator as the between-subject factor and sex and age at injury as cofactors.*


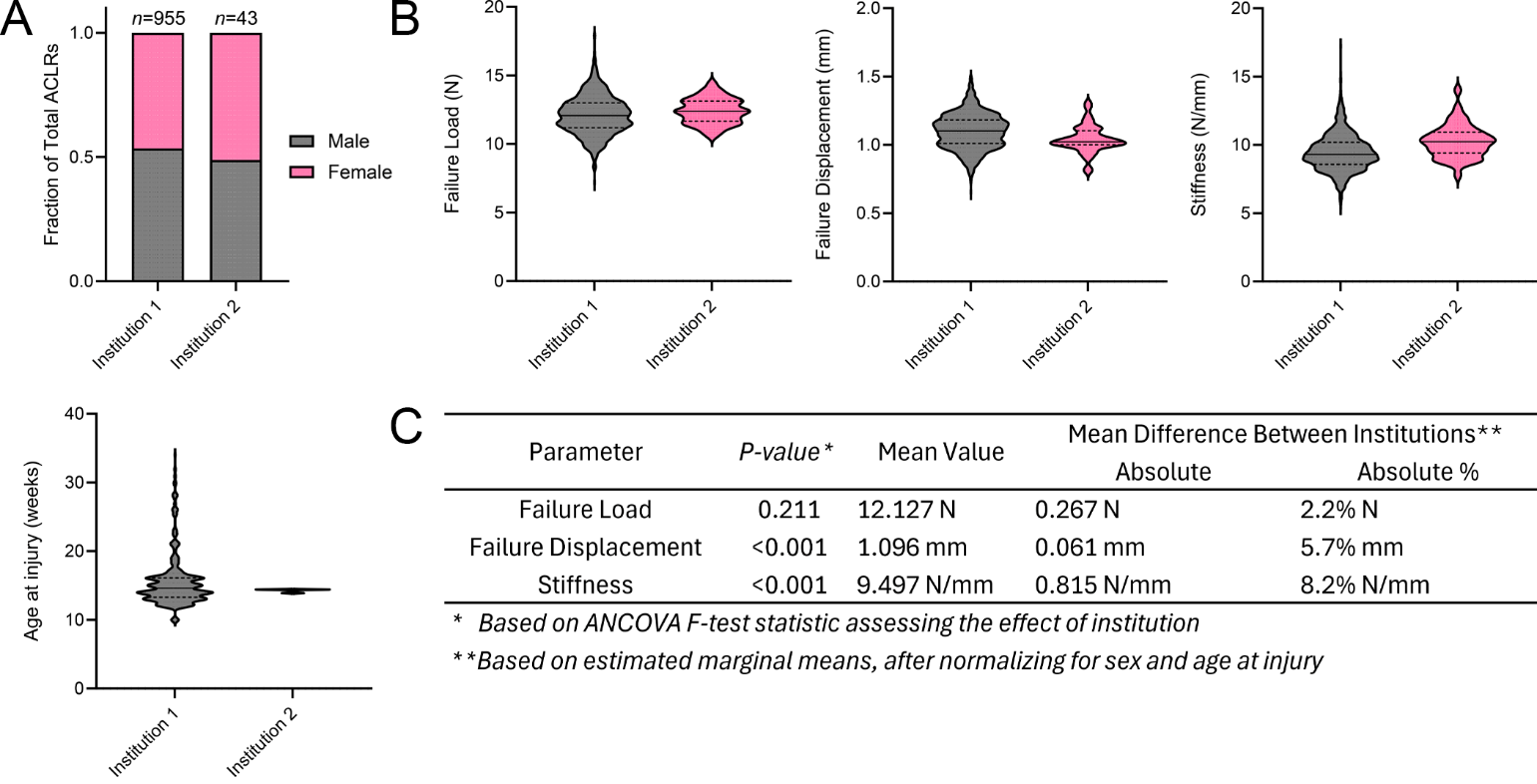


**Supplemental Figure 5.** *Reproducibility of MoJO ACL rupture outcomes between institutions. (A) Distributions of sex and age at injury among 2 institutions. (B) Mechanical parameters of ACL ruptures by institution, including failure load, failure displacement, and stiffness. (C) Statistical analysis of differences in mechanical outcomes between laboratories was assessed via ANCOVA, with institution as the between-subject factor and sex and age at injury as cofactors.*

**Supplemental Methods 1** – *Additional Information on Fixture Design*

The most critical aspect of our fixture design is the alignment between the hindpaw fixture (C) and knee fixture (D), which controls knee flexion angle and hindlimb position to facilitate ACL rupture and minimize the risk of tibial fracture. The hindpaw fixture (C) is positioned slightly cranial to the knee fixture (D) to facilitate anterior femoral subluxation, which is required for loading of the ACL. The components are aligned such that the front of the knee sits 1.5 mm cranial to the front of the ankle. Importantly, to minimize mechanical moments that may induce fixture deflection and aberrant load cell readings, the approximate mechanical axis of the applied load traveling down the tibia is in-line with the center axis of the load cell. ***Critical: Any fixture modifications cannot alter the relative position between components C and D.***

If utilized with the CellScale Univert S2 and the associated ZMSA 2 kg load cell, all fixtures can be used without modification and as described in our design. If adapting to a different mechanical testing system, components **A** and **B** require machine-specific modifications. Alternatively, new adapters amenable for a given mechanical testing system may be designed to interface with components A and B**.** Similarly, if a different load cell besides the ZMSA 2 kg load cell (part of the CellScale Univert S2 system) is used, the load cell adapters (components A and B) will require modification, or new adapters that interface with components A and B can be made.

The cylinder (E4) is only needed to raise the animal bed into the stroke range of the Univert S2, and this component can be modified in length or eliminated entirely if utilizing a different mechanical testing system. It is highly preferable to raise the animal bed towards the crosshead using an extension such as the cylinder (E4) as opposed to placing an extension on the crosshead and component A, B, C assembly. The animal bed (E1) is large enough to accommodate mice of all sizes in addition to a standard anesthesia nose cone, which can be fastened to the bed using tape or a custom fixture. To enable visualization of hindpaw positioning within the hindpaw fixture (C), this part requires two transparent plastic platens to be glued on either side of component C. If machining is not available, these platens can be hand-cut using a sharp knife from 4.5 mm thick clear acrylic. The platens are then glued to component C using cyanoacrylate (**Suppl. Fig 1C**).

The knee fixture (D) contains a 3.5-mm wide by 1.5-mm deep trough that secures the knee in the medial-lateral and cranial directions. This geometry should fit mice of all sizes, but the trough may be widened for especially large mice, ensuring that the centerline of the trough does not move. Fillets are used to round all edges of this component to avoid skin injuries.

**Supplemental Methods 2** – *Diagnosis of Successful and Unsuccessful ACL Rupture Procedures*

During the first preload and during preconditioning cycles, the joint will exhibit viscoelastic creep. A gradual reduction in creep will be observed, with relatively stable load-displacement behavior achieved by the end of the second preload. This ensures the joint is fully seated and the ACL is fully engaged during the final compressive displacement, maximizing the rate of success and a full thickness rupture. During the final compressive 1.5-mm displacement, the load response of the joint prior to ACL rupture should be highly linear. If this is not the case, the viscous response of the ligament was not completely worked out during preloading. This can be occasionally observed in large mice, or can reflect that the limb was not fully seated in the ankle and knee troughs at the start of the protocol, and thus some of the “creep” observed actually represented the ankle and knee gradually slipping further into the fixture. Incomplete pre-injury engagement of the ACL does not necessarily guarantee an unsuccessful rupture – if the rupture displacement is characteristic of a successful ACL rupture, this still represents a successful procedure.

In our hands, the success rate of this procedure is incredibly high (99.0%), but unsuccessful procedures do occur. Representative load vs time plots of unsuccessful procedures are shown in **Fig. 1G**. Occasionally, the 1.5 mm displacement may prove insufficient to induce ACL rupture, with a higher risk in larger mice (>30g). In this case, no drop in load will be observed until the crosshead reverses direction, no audible “pop” will be heard, and no/minimal tibial subluxation will be observed. This is likely caused by improper seating of the hindlimb and higher body fat.

Tibial fracture can also occur during this procedure and is a greater risk for smaller mice. Fractures typically occur in the midshaft, will produce a sharp audible sound of a different timbre than an ACL rupture, and will typically result in abrupt, *complete* unloading of the joint, without a substantial “catch” or re-loading. Suspected fractures can be assessed via manipulation of the tibia in traction and via x-ray imaging. The mouse should be kept anesthetized throughout diagnosis, and euthanized *immediately* if a fracture has occurred. If a fracture is suspected but not confirmed, the mouse should be monitored during recovery and follow-up health checks for signs of abnormal gait and excessive pain, and follow-up x-rays can be taken as needed.

We have also observed instances of tensile rupture of the proximal tibial physis. This mode of failure is the most difficult to diagnose, but tends to produce a quieter, more gradually yielding sound. We have observed that the loading curve exhibits a more gradual, ductile yield and failure compared to the abrupt failure observed in an ACL rupture. A drop in load will occur, but rather than a sharp decrease in load followed by re-loading, a more gradual, “tumbling” unloading is observed as the physis yields and epiphysis displaces from the tibia. Suspected physeal dislocation can be assessed by ranging the knee joint, as displacement can result in either reduced range of motion or a distinct “crunchiness” arising from the epiphysis and metaphysis rubbing against one another as the physis shifts – range of motion can also be assessed during health checks the following day. X-rays can be acquired, but if the epiphysis settles back in its original position, this can be difficult to diagnose. Our group missed a tibial physis rupture and identified it during histological assessment, which shows cartilaginous healing tissue and a grossly widened physis (**Suppl. Fig 2A**). µCT images demonstrate substantial extracortical ectopic bone formation in the metaphysis underneath a shifted physis (**Suppl. Fig 2B**).

**Supplemental Methods 3** – *Calibration Spring Tuning*

The average stiffness of the mouse knee joint, i.e. the ACL and associated supporting structures, in the linear region of Step 5 in this loading protocol is ~10 N/mm. The preconditioning cycles are an important part of the protocol to ensure complete seating of the knee joint in the fixture while also preparing the joint for anterior subluxation. During the preconditioning cycles (Step 2), lower stiffnesses of ~3-5 N/mm are expected. To assist in PID tuning and to perform protocol testing or training without mice, we recommend using steel compression springs with spring rates in this range. We have employed Product #9657K94 from McMaster-Carr (steel compression spring, 2.8” long, 0.625” outer diameter, 31 lbs/in spring rate, 54 lbs max load), and have found that tuning with this spring translates accurately to live mice. We recommend running the protocol on a test spring prior to each testing day, to confirm proper system response before loading live mice. We also collect longitudinal data on our spring tests to monitor system performance over time and ensure no drift occurs.

***Critical: Test each protocol or tuning parameter modification using steel springs, as recommended above, prior to using mice.***

**Supplemental Methods 4** – *Flow Cytometry of Synovial Cell Populations*

Synovia were digested using Type IV Collagenase, Liberase TM, and DNaseI for 30 mins, as previously described^15^. Cells were pre-blocked with Mouse TruStain FcX PLUS (Biolegend, clone S17011E) then stained with fluorescently conjugated antibodies: CD11b-BV510 (Biolegend, clone M1/70), CD146-BV605 (BD OptiBuild, clone ME-9F1), CD45-BV650 (Biolegend, clone 30-F11), CD31-PE/Cy7 (Biolegend, clone 390), F4/80-APC/R700 (BD Horizon, clone T45-2342), and CD3-APC/Fire750 (Biolegend, clone 17A2). Dead cells were excluded based on uptake of TO-PRO-3 iodide. Flow cytometry was performed on a BD Fortessa and data were analyzed using FlowJo software v10.
